# α-Synuclein in human cerebrospinal fluid is principally derived from neurons of the central nervous system

**DOI:** 10.1007/s00702-012-0784-0

**Published:** 2012-03-18

**Authors:** Brit Mollenhauer, Ellen Trautmann, Birgit Otte, Juliana Ng, Annette Spreer, Peter Lange, Friederike Sixel-Döring, Mansoureh Hakimi, Jean-Paul VonSattel, Robert Nussbaum, Claudia Trenkwalder, Michael G. Schlossmacher

**Affiliations:** 1 Paracelsus-Elena-Klinik, Klinikstrasse 16, 34128 Kassel, Germany; 2 Division of Neuroscience, Ottawa Hospital Research Institute, University of Ottawa, Ottawa, ON Canada; 3 Department of Neurology and Clinical Neurophysiology, Georg-August University Goettingen, Goettingen, Germany; 4 Institute of Pathology, Columbia University, New York, NY USA; 5 Division of Medical Genetics, Department of Medicine, University of California, San Francisco, San Francisco, CA USA

**Keywords:** α-Synuclein, Cerebrospinal fluid, Blood–CSF barrier, Biomarker, Choroid plexus

## Abstract

The source of Parkinson disease-linked α-synuclein (aSyn) in human cerebrospinal fluid (CSF) remains unknown. We decided to measure the concentration of aSyn and its gradient in human CSF specimens and compared it with serum to explore its origin. We correlated aSyn concentrations in CSF versus serum (Q_aSyn_) to the albumin quotient (Q_albumin_) to evaluate its relation to blood–CSF barrier function. We also compared aSyn with several other CSF constituents of either central or peripheral sources (or both) including albumin, neuron-specific enolase, β-trace protein and total protein content. Finally, we examined whether aSyn is present within the structures of the choroid plexus (CP). We observed that Q_aSyn_ did not rise or fall with Q_albumin_ values, a relative measure of blood–CSF barrier integrity. In our CSF gradient analyses, aSyn levels decreased slightly from rostral to caudal fractions, in parallel to the recorded changes for neuron-specific enolase; the opposite trend was recorded for total protein, albumin and β-trace protein. The latter showed higher concentrations in caudal CSF fractions due to the diffusion-mediated transfer of proteins from blood and leptomeninges into CSF in the lower regions of the spine. In *postmortem* sections of human brain, we detected highly variable aSyn reactivity within the epithelial cell layer of CP in patients diagnosed with a range of neurological diseases; however, in sections of mice that express only human *SNCA* alleles (and in those without any *Snca* gene expression), we detected no aSyn signal in the epithelial cells of the CP. We conclude from these complementary results that despite its higher levels in peripheral blood products, neurons of the brain and spinal cord represent the principal source of aSyn in human CSF.

## Introduction

The pathological hallmarks of Parkinson disease (PD) include progressive cell loss of selective nuclei in the nervous system and the formation of intracellular aggregates that contain insoluble, wild-type α-synuclein (aSyn) (Spillantini et al. 1997). The clinical phenotype of autosomal dominant PD parallels aSyn gene (*SNCA*) dosage with a more severe phenotype observed in the cases of locus multiplication (Singleton et al. 2003). The quantification of the 140 amino acid-long, 16–17 kDa aSyn protein in biological fluids has been proposed as a biomarker candidate (Mollenhauer et al. 2010). aSyn has been shown to be present in cerebrospinal fluid (CSF) in low picogram/microliter amounts; its levels in serum and plasma are up to tenfold and in whole blood up to 10,000-fold higher (Mollenhauer et al. 2010) due to the fact that aSyn is also abundantly expressed in the hematopoietic system (Scherzer et al. 2008).

The production and hydrodynamics of human CSF are complex and vary considerably depending on the level within the neuroaxis. The three main sources for CSF constituents comprise: one, the metabolism of neural cells in the central nervous system (CNS); two, diffusion of constituents from circulating blood into CSF [even in the absence of any trauma (Hong et al. 2010; Mollenhauer et al. 2011)]; and three, the secretion by the epithelial cells that reside within the brain’s choroid plexus (CP) (Chodobski and Szmydynger-Chodobska 2001). CSF volume and its continuous renewal are predominantly regulated by CP activity. An estimated 80% of all CSF proteins are blood-derived (with albumin being the most abundant); their concentrations in the nervous system increase in the subarachnoid space from rostral to caudal levels due to diffusion gradients that emanate from blood vessels intersecting with the flow of CSF (Reiber 2003). A rise from rostrum to cauda is also observed for certain constituents that are secreted by leptomeningeal structures (e.g., β-trace protein) (Reiber 2003). In the case of abnormally low CSF flow rates, which result in increasing concentrations of blood-derived proteins in lumbar CSF fractions, the concentration of brain-derived proteins [e.g., neuronspecific enolase (NSE)] usually remains constant (Reiber 2003). Because albumin is solely synthesized in the liver, the quotient of its CSF-to-serum concentrations (referred to as Q_albumin_) reflects an indirect measure of blood–CSF barrier function in vivo.

This study was designed to investigate whether CSF aSyn is mainly brain or blood derived. To this end, we compared aSyn concentrations in CSF versus serum (Q_aSyn_) with Q_albumin_ in 42 patients with various neurological diseases. Next, we quantified aSyn levels in patients with normal blood–CSF barrier function and compared them to specific brain-derived (e.g., NSE), blood-derived (e.g., albumin), and leptomeninges-derived (e.g., β-trace) proteins in serially collected CSF samples, to examine their rostro-caudal concentration gradients. Furthermore, we also probed whether CP tissue contained aSyn reactivity.

## Methods

We collected CSF and serum from patients with different neurological conditions. These 42 participants underwent lumbar puncture (LP) for routine diagnostic purposes (Table 1) to determine the presence (or absence) of acute inflammatory disease, acute hemorrhage and suspected Lyme disease (*n* = 31); to exclude chronic inflammatory diseases (*n* = 3); to determine carcinomatosis of the leptomeninges in the case of a known primary tumor (*n* = 4), and to carry out CSF volume reduction in patients with normal pressure hydrocephalus (NPH; *n* = 4). All study participants were enrolled at the Department of Neurology at Goettingen University, Germany. Samples were collected and processed as published (Mollenhauer et al. 2010). We further examined samples that featured: a normal (<5/μl) white blood cell count; the absence of intra-thecal immunoglobulin production (as determined by the absence of oligoclonal bands); normal CSF tau protein, and no LP-induced contamination by blood (determined by a red blood cell count of <50/μl). CSF aSyn and serum aSyn (diluted at 1:15) concentrations were measured by enzyme-linked immunoabsorbent assay (ELISA) as published (Mollenhauer et al. 2011).

**Table 1:** Demographic data of enrolled patients of both cohorts

	Set 1	Set 2
*N*	42	5
Sex (male/total)	0.5	0.6
Age (years)	58 ± 21	76 ± 4
Mean ± standard deviation (range)	(19–93)	(69–79)

In a second set of study participants we collected CSF from five patients at the Paracelsus-Elena-Klinik, Kassel, Germany. All patients suffered from NPH as determined by magnetic resonance imaging and according to the established clinical criteria (Relkin et al. 2005) (Table 1). We performed volume reduction by LP on each patient and serially collected seven CSF fractions (5 ml each) that were processed as published (Mollenhauer et al. 2010). LP was carried out at the L2/L3 level in the sitting position, between 8 and 9 a.m. with the patient having fastened for 12 h. In each fraction we analyzed CSF aSyn (Mollenhauer et al. 2011), NSE (Schaarschmidt et al. 1994) and β-trace protein (Tumani et al. 1998), as described. Total protein and albumin levels were measured by nephelometry (Dade Behring/Siemens Healthcare Diagnostics).

Immunohistochemistry of *postmortem* brain sections containing CP structures was carried out following formalin fixation and paraffin embedding with anti-aSyn antibodies, as published (Louis et al. 2005; Schlossmacher et al. 2002). In parallel, sections from mouse brain transgenic for four Ala53Thr-containing human *SNCA* alleles (but no mouse *Snca*) and *Snca*-null mice without any endogenous *Snca* and no transgene expression [engineered as described (Kuo et al. 2011)] were processed by the same protocol. Polyclonal (hSA5.1; Open Biosystems Inc) and monoclonal (12–1; Epitomics Inc) antibodies to wild-type, full-length human aSyn were raised in goat and rabbit, respectively, purified and characterized (Cullen et al. 2009; Mollenhauer et al. 2008).

The study was carried out in accordance with the Declaration of Helsinki and with informed written consent provided by all patients or their next of kin. The study was approved by the ethics committee at the University of Goettingen (Germany).

### Analysis

**Fig. 1:**
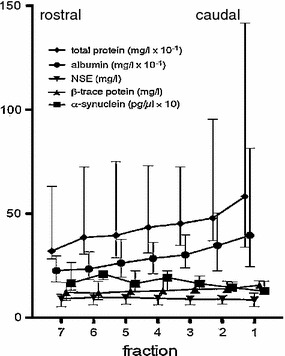
Median (and interquartile range) values for CSF total protein (in mg/dl), albumin (in mg/dl), neuron specific enolase (NSE) (in mg/l), β-trace-protein (mg/l) and α-synuclein (aSyn) (in pg/μl ×10) in all seven fractions

Possible correlations between the concentrations of CSF/serum aSyn to CSF/serum albumin (i.e., Q_albumin_) were analyzed according to Pearson. Results for aSyn, NSE, β-trace, albumin and total protein values in CSF are demonstrated in Table 2 and Fig. 1. Statistical analyses (non-parametric) of the data were performed to assess the characteristics in CSF vials 5–7 (cisternal fractions) and 1–4 (lumbar fractions) for each described variable using the trend test from Page (Page 1963). The Page’s L trend test examined whether the levels are ordered in a specific order/sequence. For all tests, the α-level of significance was set at 5 %. Furthermore, effect sizes (Hedge’s g) were calculated (Ray and Shadish 1996). A correction formula for small sample bias was also applied (Hedges and Olkin 1985).

**Table 2:** Quantification results including mean levels in seven fractions (F1 lower F7 upper position) for CSF α-synuclein (aSyn), total protein, albumin, neuron specific enolase (NSE) and β-trace protein

Fractions	α-Synuclein (pg/μl)	Total protein (mg/l)	Albumin (mg/l)	NSE (mg/l)	β-Trace protein (mg/l)
F7	1.92	387	224	9.49	12.66
F6	1.88	398	226	9.21	12.25
F5	1.91	436	255	9.48	12.86
F4	1.91	449	266	9.30	13.68
F3	1.79	468	290	8.89	14.20
F2	1.69	526	321	9.09	13.94
F1	1.66	634	401	8.41	14.02
Rostrocaudal ratio	1.16	0.61	0.56	1.13	0.90

## Results

### CSF α-synuclein in subjects with known blood–CSF barrier function

**Fig. 2:**
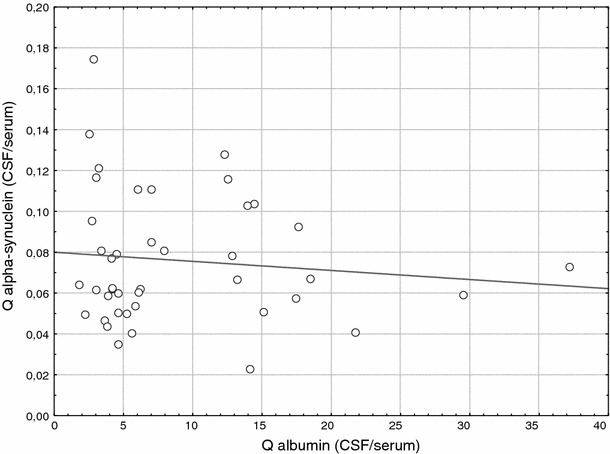
Correlation of Q_aSyn_ and Q_albumin_ (CSF/serum) (*p* > 0.05)

CSF specimens analyzed from 42 patients showed a mean Q_albumin_ of 8.90 ± 7.7 × 10^−3^ (mean ± standard deviation; SD) (range 1.8–37.2 × 10^−3^). CSF aSyn levels ranged from 1.29 to 2.97 pg/μl (mean ± SD; 1.99 ± 0.39). Serum aSyn levels ranged from 14.33 to 67.47 pg/μl (mean 29.54 ± 10.79), and the mean CSF/serum aSyn (Q_aSyn_) was calculated as 0.08 ± 0.032. The coefficients of variation (CV) for aSyn values measured 19.6% in CSF, 36.5% in serum, and the CV for Q_aSyn_ was 40%. Importantly, when examining Q_aSyn_ together with Q_albumin_ values, we recorded no significant correlation (−0.109; *p* = 0.493; Fig. 2).

### CSF α-synuclein and other protein values from rostro-caudal fractions

CSF aSyn levels across all fractions examined ranged from 1.17 to 2.64 pg/μl (mean 1.70 ± 0.36) in specimens from five NPH patients that were analyzed. Q_albumin_ in these cases ranged between 5.4 and 7.8 × 10^−3^. Across the 35 ml of CSF collected in our NPH patients, aSyn concentrations showed a slight increase that initially paralleled the proximity of the sample collected toward the brain (and as reflected by increasing fraction numbers in the first 20 ml collected), which represented a trend without statistical significance (*L* = 130; *p* > 0.05). The levels of exclusively neuron-derived NSE remained stable throughout (*L* = 134; *p* > 0.05; Table 2; Fig. 1). In contrast, blood-derived albumin (*L* = 145.5; *p* < 0.05) and total protein (*L* = 139; *p* < 0.05) decrease significantly in the rostral fractions (#5 to #7, i.e., in the last 15 ml of each LP volume collected; Table 2; Fig. 1) compared to caudal fractions (#1 to #4, i.e., corresponding to the first 20 ml collected), as expected from the published literature (Sullivan et al. 2006). Albeit to a lesser extent, leptomeninges-derived β-trace protein also showed a mild decrease in its concentration with progression from the lumbar region toward the cranial compartment (*L* = 138.5; *p* < 0.05). Figure 1 shows the median values for each fraction and for each protein.

### Choroid plexus epithelium is an unlikely source of α-synuclein

**Fig. 3:**
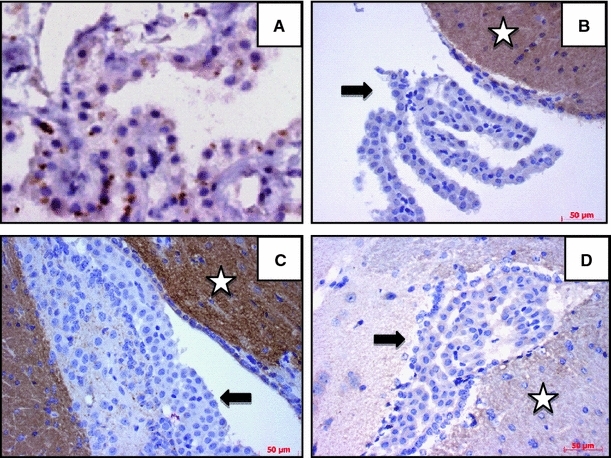
Probing for α-synuclein (aSyn) reactivity in mammalian choroid plexus. Formalin-fixed, paraffin-embedded sections of *postmortem* human brain are shown, including from a 65-year-old man with progressive supranuclear palsy (**a**), and of ‘humanized *SNCA*’-transgenic mouse brain (**b**, **c**) with a segment of the 4th ventricle (**a**, **b**) or lateral ventricle (**c**). All sections contain choroid plexus (CP) epithelium and were probed for aSyn reactivity with anti-aSyn antibodies by routine immunohistochemistry and counterstained with hematoxyline. Cortical sections from a *Snca*-null mouse (**d**) were processed in parallel. Antibodies used in **a**, **b** and **d**: goat-anti-human aSyn (hSA5.1); in **c**, monoclonal rabbit antibody to human aSyn (12–1). Representative specimens from human autopsy material (*n* = 5), *hSNCA* ^A53T^-transgenic mice (*n* = 4) and *Snca*-null animals (*n* = 2) are shown. *Arrows* identify unlabelled epithelial cells of CP; in contrast, *stars* denote the robust expression of aSyn in the neuropil of *SNCA*-expressing mice

The intra-ventricular network of the CP represents the predominant site for CSF volume production in vivo. To also explore CP as a possible source for CSF aSyn, we first examined *postmortem* human brain sections by routine immunohistochemistry (IHC). We unexpectedly detected highly variable aSyn reactivity within the epithelial cell layer of human CP (Fig. 3a). When comparing sections from several diagnostic categories, we saw considerable differences in the staining intensity of adult CP epithelium among cases with synucleinopathy [PD, dementia with Lewy bodies (DLB)], progressive supranuclear palsy, Alzheimer’s and Huntington disease and aged control subjects (Fig. 3a; data not shown), but did not record a correlation between aSyn reactivity in CP epithelium and the final diagnosis. To determine whether the aSyn signal in *postmortem* CP was reflective of physiological metabolism in vivo, we decided to examine brain sections of “humanized” *SNCA*-transgenic mice (Kuo et al. 2011) versus animals deficient in murine (and human) aSyn using the same IHC protocol. There, we detected no aSyn reactivity in the CP epithelium-containing sections of *SNCA*-transgenic mice, wild-type animals and *Snca*-null mice (Fig. 3b–d; data not shown). In contrast, CNS structures including neurons and presynaptic terminals (as well as intravascular erythrocytes) showed strong aSyn immunoreactvity, as expected from the corresponding genotype (Fig. 3b, c; data not shown). We concluded from these findings that CP, a highly vascularized organ within the brain, represents an unlikely source for the constitutive release of aSyn into CSF in vivo; however, a possible uptake of aSyn from CSF by CP cells *postmortem* could not be excluded.

## Discussion

To elucidate the source of aSyn in CSF, we measured its levels in CSF and serum from a cohort with variable Q_albumin_ values (Blennow et al. 1993; Reiber 2003) and in a second, small cohort of NPH patients, where we measured CSF aSyn together with several proteins from different CNS sources. Our collective results suggest that CSF aSyn is predominantly derived from structures and functions associated with the brain rather than from peripheral blood products.

CSF aSyn has been discussed as a possible biomarker for aSyn-related disorders such as PD, DLB and multiple system atrophy (MSA) (Mollenhauer et al. 2011) and reviewed in (Mollenhauer et al. 2010); its presence in normal human CSF has been previously confirmed by mass spectrometry (Mollenhauer et al. 2008). The majority (but not all) of the CSF aSyn quantification studies conducted over the last 6 years have shown a decrease of total aSyn levels in CSF from PD, DLB and MSA patients, as measured by ELISA and Luminex platforms [reviewed in: (Mollenhauer et al. 2010, 2011)]. These biomarker exploration efforts raised—among other issues—the question of the possible sources of CSF aSyn, which theoretically include the following: (1) physiological exocytosis by neural cells of the brain and spinal cord (Lee et al. 2005; Mollenhauer et al. 2008); (2) secretion by the epithelial cells of the CP; (3) release by cells of the ependymal lining of the brain’s ventricular system; (4) secretion by endothelial cells of the brain’s vasculature; (5) normal filtration of peripheral plasma through the brain’s capillary system (or following a compromise in the integrity of the blood–CSF barrier); (6) diffusion and/or entry of blood products in anatomically privileged sites without effective blood–CSF barrier; and lastly, (7) physiological attrition of neural cells as well as neuronal injury in the CNS.

Because aSyn is highly abundant in blood cells, especially in erythrocytes, discrepant findings across cross-sectional studies have raised the possibility of divergence due to artificial blood contamination, which occurs in 10–20% of routine LPs (Mollenhauer et al. 2010, 2011). However, in most (but not all) published CSF aSyn quantification studies, the possibility of blood contamination as the source of CSF aSyn has been addressed and largely excluded [reviewed in (Mollenhauer et al. 2010)].

In examining the influence of the blood–brain barrier on CSF and serum aSyn levels from neurological controls, we found no correlation between the concentration of CSF aSyn and Q_aSyn_ with Q_albumin_. Therefore, we concluded that CSF aSyn levels were not controlled by the blood–CSF barrier under physiological conditions. However, larger cohorts comprising patients with various inflammatory conditions will need to be examined (e.g., multiple sclerosis) to discern the effect of aSyn distribution under pathological blood–CSF barrier conditions.

Due to the protective role of the barrier function on CNS integrity, we also calculated the coefficients of variation (CV) as a reflection of biological variability. The CV value for aSyn in CSF was smaller than that for serum aSyn. The latter observation provided independent (albeit indirect) support for our conclusion that under normal conditions the majority of aSyn species detected in CSF was unlikely to be blood borne; if it would have been predominantly derived from blood, the CV of CSF aSyn values would have been expected to be at least equal—if not larger—than those of serum aSyn. Another possible explanation for the different CV values is that our assay could have captured CSF aSyn more readily than serum aSyn (for example due to protein complex formation and thus altered antibody accessibility), but this scenario seems unlikely given the significantly larger amounts of aSyn recorded by our assay in all serum samples than in CSF (Mollenhauer et al. 2010, 2011).

To further examine aSyn levels along the rostro-caudal flow of CSF, we collected a total of 35 ml in seven serial fractions in all NPH patients. The lumbosacral CSF volume is highly variable among individual patients and is estimated to contain 35.8 ± 10.9 ml with a total range from 10.6 to 61.3 ml in adult humans (Sullivan et al. 2006). With the LP performed at the L2/L3 level in all study participants, we inferred that fractions #1–4 contained CSF from the caudal subarachnoidal space where it supports spinal cord suspension (and function), whereas fractions #5–7 represented CSF derived from space closer to (and above) the craniocervical juncture following direct contact with the brain. Our analysis revealed the expected rostro-caudal increase of plasma-derived proteins (i.e., total protein and albumin), which is thought to reflect a longer transit time of CSF to the lumbar spine region and a higher proportion of diffusion-mediated transfer of proteins from blood to CSF (Reiber 2003). In our study, β-trace protein concentrations also increased in a rostro-caudal gradient, as expected from studies that simultaneously analyzed ventricular and lumbar CSF samples (Reiber 2001, 2003); β-trace protein is mainly released by leptomeningeal structures. In contrast, the neuron-derived protein NSE showed the expected rostro-caudal stability in our hands, possibly even a slight reduction for the concentrations recorded in lumbar fractions #1–4. These collective findings strongly supported the conclusions that these seven fractions indeed represented different levels of CSF collected from the lumbar as well as cisternal regions of subarachnoid space. The concentration behavior of our analyte of interest, total aSyn, behaved most closely to that of NSE en route from the rostrum of the brain to the lumbosacral space. This decreasing rostro-caudal gradient is, therefore, in support of a concept that sees the brain as the most important source of CSF aSyn followed by nervous system structures below the medulla.

A decreasing rostro-caudal gradient for CSF aSyn concentrations was also reported by Hong and colleagues, who analyzed three fractions in 45 control subjects showing a trend for decreased CSF aSyn in lower # fractions (Hong et al. 2010). Although the Hong et al. CSF samples were not centrifuged after their collection, therefore, raising the theoretical possibility of blood and epithelial cell contamination (which could have influenced their findings), our results confirm and expand the conclusions reached by these and other authors exploring the source of extracellular aSyn in human brain (Emmanouilidou et al. 2011).

Our study on CSF aSyn is limited by its small sample size and a selection bias towards patients with non-neurodegenerative conditions. In future studies, patients with neurodegenerative and inflammatory illnesses as well as medically healthy participants have to be analyzed. Nevertheless, our results support the concept of a source of CSF aSyn that is predominantly (but not exclusively) associated with nervous system structure and function under physiological conditions, and is less likely to be blood borne. Our results do not exclude the possibility that this distribution is markedly altered in diseases associated with significant neuronal lysis (e.g., due to prion disease) (Mollenhauer et al. 2008).

Our finding of variable immunoreactivities for aSyn within the epithelium of CP in human brain (but not mouse brain) sections could indicate a relevant site for aSyn uptake *postmortem* from circulating CSF [as reported for example for amyloid-β-protein (Crossgrove et al. 2005) (Wolburg and Paulus 2010)] in some patients. However, our findings in genetically engineered mice make de novo gene transcription in CP and secretion (or filtration of aSyn from plasma into CSF by CP epithelial cells) very unlikely. Alternatively, technical reasons (such as those related to possible antibody cross-reactivity with unknown antigens) when processing human versus mouse brain could also explain the observed species difference. Lastly, in future studies we will revisit whether this variable immunoreactivity for aSyn in human CP may be related to the final diagnosis of the patient. To date, we have not yet observed such a correlation.

Understanding the sources of aSyn, delineating the molecular events underlying its presumed exocytosis by cells into the interstitial, ventricular and subarachnoid spaces of the CNS, and cataloging its modified variants represent pivotal tasks for our field in the future. They are important in the identification of the unknown function of aSyn in CSF, its main degradation pathways in intra- and extracellular compartments (including in CSF), and its possible uptake by neural, ependymal and epithelial cells from circulating CSF. These related topics have major implications for the pathogenesis, the diagnosis and possibly, the progression of several, currently incurable synucleinopathy disorders of the brain including PD, MSA and DLB (Desplats et al. 2009; Hong et al. 2010; Kramer and Schulz-Schaeffer 2007; Mollenhauer et al. 2011; Volpicelli-Daley et al. 2011).

## References

[CR1] Blennow K, Fredman P, Wallin A, Gottfries CG, Karlsson I, Langstrom G, Skoog I, Svennerholm L, Wikkelso C (1993). Protein analysis in cerebrospinal fluid. II. Reference values derived from healthy individuals 18–88 years of age. Eur Neurol.

[CR2] Chodobski A, Szmydynger-Chodobska J (2001). Choroid plexus: target for polypeptides and site of their synthesis. Microsc Res Tech.

[CR3] Crossgrove JS, Li GJ, Zheng W (2005). The Choroid Plexus Removes {beta}-Amyloid from Brain Cerebrospinal Fluid. Exp Biol Med.

[CR4] Cullen V, Lindfors M, Ng J, Paetau A, Swinton E, Kolodziej P, Boston H, Saftig P, Woulfe J, Feany MB, Myllykangas L, Schlossmacher MG, Tyynela J (2009). Cathepsin D expression level affects alpha-synuclein processing, aggregation, and toxicity in vivo. Mol Brain.

[CR5] Desplats P, Lee HJ, Bae EJ, Patrick C, Rockenstein E, Crews L, Spencer B, Masliah E, Lee SJ (2009). Inclusion formation and neuronal cell death through neuron-to-neuron transmission of alpha-synuclein. Proc Natl Acad Sci USA.

[CR6] Emmanouilidou E, Elenis D, Papasilekas T, Stranjalis G, Gerozissis K, Ioannou PC, Vekrellis K (2011). Assessment of alpha-synuclein secretion in mouse and human brain parenchyma. PLoS ONE.

[CR7] Grouper S, Walker MT, Parrish TB, McCarthy RJ, Wong CA (2006). Lumbosacral cerebrospinal fluid volume in humans using three-dimensional magnetic resonance imaging. Anesth Analg.

[CR8] Hedges LV, Olkin I (1985). Statistical methods for meta-analysis.

[CR9] Hong Z, Shi M, Chung KA, Quinn JF, Peskind ER, Galasko D, Jankovic J, Zabetian CP, Leverenz JB, Baird G, Montine TJ, Hancock AM, Hwang H, Pan C, Bradner J, Kang UJ, Jensen PH, Zhang J (2010). DJ-1 and alpha-synuclein in human cerebrospinal fluid as biomarkers of Parkinson’s disease. Brain.

[CR10] Kramer ML, Schulz-Schaeffer WJ (2007). Presynaptic alpha-synuclein aggregates, not Lewy bodies, cause neurodegeneration in dementia with Lewy bodies. J Neurosci.

[CR11] Kuo YM, Li Z, Jiao Y, Gaborit N, Pani AK, Orrison BM, Bruneau BG, Giasson BI, Smeyne RJ, Gershon MD, Nussbaum RL (2011). Extensive enteric nervous system abnormalities in mice transgenic for artificial chromosomes containing Parkinson disease-associated alpha-synuclein gene mutations precede central nervous system changes. Hum Mol Genet.

[CR12] Lee HJ, Patel S, Lee SJ (2005). Intravesicular localization and exocytosis of alpha-synuclein and its aggregates. J Neurosci.

[CR13] Louis ED, Honig LS, Vonsattel JP, Maraganore DM, Borden S, Moskowitz CB (2005). Essential tremor associated with focal nonnigral Lewy bodies: a clinicopathologic study. Arch Neurol.

[CR14] Mollenhauer B, Cullen V, Kahn I, Krastins B, Outeiro TF, Pepivani I, Ng J, Schulz-Schaeffer W, Kretzschmar HA, McLean PJ, Trenkwalder C, Sarracino DA, Vonsattel JP, Locascio JJ, El-Agnaf OM, Schlossmacher MG (2008). Direct quantification of CSF alpha-synuclein by ELISA and first cross-sectional study in patients with neurodegeneration. Exp Neurol.

[CR15] Mollenhauer B, El-Agnaf OM, Marcus K, Trenkwalder C, Schlossmacher MG (2010). Quantification of alpha-synuclein in cerebrospinal fluid as a biomarker candidate: review of the literature and considerations for future studies. Biomark Med.

[CR16] Mollenhauer B, Locascio JJ, Schulz-Schaeffer W, Sixel-Doring F, Trenkwalder C, Schlossmacher MG (2011). alpha-Synuclein and tau concentrations in cerebrospinal fluid of patients presenting with parkinsonism: a cohort study. Lancet Neurol.

[CR17] Page EB (1963). Ordered hypothesis for multiple treatments: A significance test for linear ranks. J Am Stat Assoc.

[CR18] Ray JW, Shadish WR (1996). How interchangeable are different estimators of effect size?. J Consult Clin Psychol.

[CR19] Reiber H (2001). Dynamics of brain-derived proteins in cerebrospinal fluid. Clin Chim Acta.

[CR20] Reiber H (2003). Proteins in cerebrospinal fluid and blood: barriers, CSF flow rate and source-related dynamics. Restor Neurol Neurosci.

[CR21] Relkin N, Marmarou A, Klinge P, Bergsneider M, Black PM (2005). Diagnosing idiopathic normal-pressure hydrocephalus. Neurosurgery.

[CR22] Schaarschmidt H, Prange HW, Reiber H (1994). Neuron-specific enolase concentrations in blood as a prognostic parameter in cerebrovascular diseases. Stroke.

[CR23] Scherzer CR, Grass JA, Liao Z, Pepivani I, Zheng B, Eklund AC, Ney PA, Ng J, McGoldrick M, Mollenhauer B, Bresnick EH, Schlossmacher MG (2008). GATA transcription factors directly regulate the Parkinson’s disease-linked gene alpha-synuclein. Proc Natl Acad Sci USA.

[CR24] Schlossmacher MG, Frosch MP, Gai WP, Medina M, Sharma N, Forno L, Ochiishi T, Shimura H, Sharon R, Hattori N, Langston JW, Mizuno Y, Hyman BT, Selkoe DJ, Kosik KS (2002). Parkin localizes to the Lewy bodies of Parkinson disease and dementia with Lewy bodies. Am J Pathol.

[CR25] Singleton AB, Farrer M, Johnson J, Singleton A, Hague S, Kachergus J, Hulihan M, Peuralinna T, Dutra A, Nussbaum R, Lincoln S, Crawley A, Hanson M, Maraganore D, Adler C, Cookson MR, Muenter M, Baptista M, Miller D, Blancato J, Hardy J, Gwinn-Hardy K (2003). Alpha-Synuclein locus triplication causes Parkinson’s disease. Science.

[CR26] Spillantini MG, Schmidt ML, Lee VM, Trojanowski JQ, Jakes R, Goedert M (1997). Alpha-synuclein in Lewy bodies. Nature.

[CR27] Tumani H, Nau R, Felgenhauer K (1998). Beta-trace protein in cerebrospinal fluid: a blood-CSF barrier-related evaluation in neurological diseases. Ann Neurol.

[CR28] Volpicelli-Daley LA, Luk KC, Patel TP, Tanik SA, Riddle DM, Stieber A, Meaney DF, Trojanowski JQ, Lee VM (2011). Exogenous alpha-synuclein fibrils induce Lewy body pathology leading to synaptic dysfunction and neuron death. Neuron.

[CR29] Wolburg H, Paulus W (2010). Choroid plexus: biology and pathology. Acta Neuropathol.

